# Computed Tomography Coronary Angiography Is Feasible and Reliable for Proximal Coronary Segment Interpretation in Patients with Elevated Body Mass Index

**DOI:** 10.3390/jcdd11120400

**Published:** 2024-12-11

**Authors:** Anthony Salib, Michael Hay, Rahul Muthalaly, Timothy Abrahams, Nushrat Sultana, Raj Kanna, Ravi Rao, Akira Abe, John Bastwrous, Emma Aldous, Huong Tu, Sarang Paleri, Sheran Vasanthakumar, Alisha Patel, Rhea Nandurkar, Adam Brown, Andrew Lin, Nitesh Nerlekar

**Affiliations:** 1 Faculty of Medicine, Monash University, Wellington Rd, Melbourne 3800, Australiaadam.brown@monash.edu (A.B.); andrew.lin@monash.edu (A.L.); 2 Monash Cardiovascular Research Centre, Victorian Heart Hospital, Blackburn Rd, Melbourne 3800, Australia; 3 Independent Researcher, Perth 6000, Australia; 4 Independent Researcher, Melbourne 3800, Australia; 5 Baker Heart and Diabetes Institute, Melbourne 3004, Australia

**Keywords:** obesity, CTCA, CAD, proximal coronary segment

## Abstract

Computed tomography coronary angiography (CTCA) is under-utilised in detecting coronary artery disease (CAD) in obese patients due to concerns about non-evaluable testing. We hypothesise that these concerns are predominantly related to smaller and branch coronary vessels, and CTCA remains adequate for proximal segment stenosis interpretation, which has significant clinical implications. This retrospective cohort study, on consecutive patients referred for CTCA for suspected CAD, grouped patients by body mass index. A 4-point Likert scale assessed image quality, with any poorly visualised segment at the per-patient level resulting in the CTCA being subsequently analysed for proximal coronary artery segment evaluability. Of the 703 patients, 93.5% of the studies were fully evaluable. Patients with a BMI ≥ 40, diabetic patients, and patients with an elevated acquisition heart rate were associated with suboptimal studies. Of the 46 suboptimal studies, 163/182 (90%) of proximal segments were fully evaluable. Non-evaluable segments were derived from seven patients (one with a BMI ≥ 40). Reasons for proximal segment non-evaluability were predominantly due to calcific blooming (12/19 segments). While CTCA may be less reliable for distal and side-branch artery evaluation in obese patients, it remains highly evaluable for stenosis severity of the proximal main coronary segments, which carries prognostic significance. It may therefore be considered a suitable non-invasive anatomic test for patients, regardless of BMI.

## 1. Introduction

Coronary artery disease (CAD) is the leading cause of death in the Western world [1] The incidence of global obesity has almost tripled since 1975, and approximately 20% of the cardiovascular disease burden can be attributed to obesity, with one in three Australians being obese [2,3]. Several studies have reported an association between increased body mass index (BMI, kg/m^2^), CAD, and major adverse cardiovascular events (MACE) [4,5,6]. However, CAD assessment in this high-risk group is complicated by challenges in traditional methods such as echocardiography, electrocardiography, and stress testing. Whilst invasive coronary angiography (ICA) is the gold standard, computed tomography coronary angiography (CTCA) offers a safer, non-invasive alternative for anatomical CAD assessment.

The high sensitivity and negative predictive value of CTCA has resulted in its use as a gatekeeper investigation prior to ICA [7] However, CTCA quality and interpretation are negatively influenced by increasing adiposity, and obesity has been previously thought to be a relative contraindication to CTCA [8]. Increased chest wall tissue increases CT noise, defined as grainy pixel distortion that reduces interpretability. Increased radiation delivery can reduce CT noise, although this carries its own associated risks. Differences in circulatory distribution exist in overweight individuals, often leading to poorly opacified coronary vessels upon contrast injection [8]. Additionally, table weight limits and gantry size may also present obstacles for the CTCA imaging of people with obesity. Consequently, clinician hesitancy limits CTCA use for this group, leading to equipoise towards the assessment of CAD in those affected by obesity and clinician bias when choosing the appropriate coronary assessment for this high-risk group. 

Given the natural tapering of coronary vessels towards distal branches and the tendency for high-risk plaque to cluster in proximal segments, thorough evaluation of proximal coronary segments is critical in anatomic diagnostic testing [9]. While obesity often impairs complete CTCA interpretability, limited data exist on the interpretability of the proximal segments alone. In this study, we aimed to determine the diagnostic interpretability of CTCA across various body mass index (BMI) categories, identify predictors for suboptimal studies, and assess the diagnostic yield of image interpretation restricted to the proximal coronary segments in scans with suboptimal image quality.

## 2. Materials and Methods

Study Design: The study was approved by the Human Research Ethics Committee in accordance with the ethical guidelines of the 1975 Declaration of Helsinki with a waiver for informed consent. De-identified participants were retrospectively and consecutively recruited from a large cardiology practice in Perth, Western Australia (Global Cardiology). The inclusion criteria consisted of having undergone CTCA at this clinic between July 2022 and July 2023. The exclusion criteria included a history of known atherosclerotic disease (i.e., diagnosed CAD, peripheral vascular disease, or a cerebrovascular accident), a history of revascularisation (percutaneous coronary intervention or coronary artery bypass graft surgery), or participants aged <40 or ≥80 years. The participants were grouped into BMI categories of BMI 18.5–24.9, BMI 25–39.9, and BMI ≥ 40. 

Patient characteristics were obtained through electronic medical records, and included age, sex, BMI, cardiovascular risk factors (hypertension, diabetes, dyslipidaemia), and cardiovascular medications (cardio-selective antihypertensives agents, antihyperglycaemic agents, and statins). A positive smoking status was established to include both current and former smoking, and a family history of premature CAD was established based on at least one first-degree relative <60 or a second-degree relative <55 years old. 

CT Protocol. A 320-detector row system (AquilionOne, Toshiba Medical Systems, Tokyo, Japan) was used to perform all CTCAs using a standardised departmental protocol. Patients received oral metoprolol pre-medication (50 mg in divided doses), and heart rate was measured on arrival. Intravenous metoprolol was liberally used to aim for a heart rate of <60 bpm. Nitroglycerin was administered 1 min prior to contrast injection. A bolus of 100% Iohexal was administered at 6 mL/second followed by a 50 mL normal saline chaser. Scanning was manually triggered when peak contrast enhancement was observed in the left ventricle and with no enhancement observed in the right ventricle. Tube current was determined by automatic exposure control on the basis of X-ray attenuation in the initial scout images and the reconstruction kernel. Tube potential was manually altered by the radiographer, with a default of 100 kVp (range: 80–120 kVp). Scans were performed with prospective electrographic triggering, using 70–85% of the phase window (increasing to 30–90% if heart rate increased, despite maximal intravenous metoprolol administration). Images were reconstructed with a 512 × 512 matrix, 0.5 mm thick sections, and the use of adaptive iterative dose reduction. CTCA images were co-reported by a CT-trained cardiologist and radiologist in the same sitting, both with at least 10 years of clinical experience. A digital platform (Vitrea FX 2.0, Vital Images, Minnetonka, MN, USA) was used to review the phase with the best image quality after reconstruction. The Standardised Society of Cardiovascular Computed Tomography 18-segment model was used to quantify all segments with a ≥1.5 mm diameter [10]. Grading of stenosis severity was according to the Society of Cardiovascular Computed Tomography Guidelines. Greater than 50% luminal diameter reduction was defined as an obstructive stenosis. 

**Figure 1:**
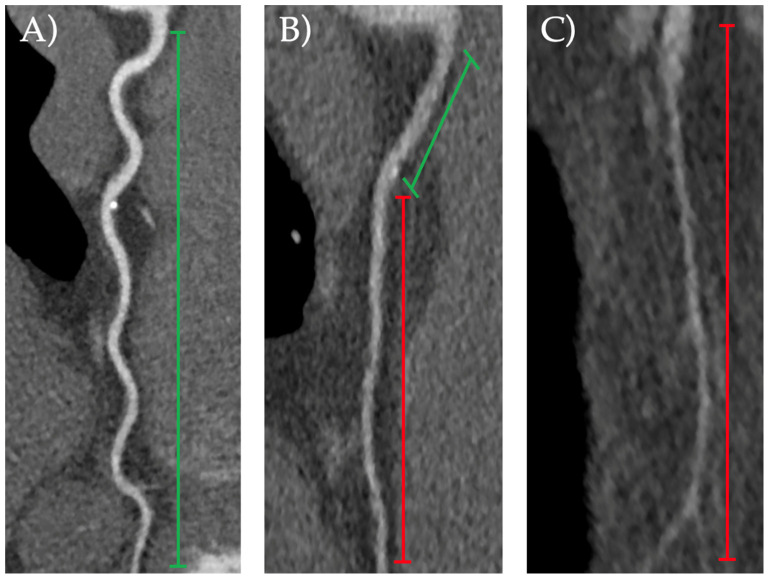
Examples of: (**A**) fully evaluable vessel; (**B**) evaluable proximal segment, with suboptimal distal vessel visualisation; (**C**) full-vessel suboptimal visualisation. Green lines indicate evaluable coronary segments, red lines indicate suboptimal coronary segment visualisation.

**Figure 2:**
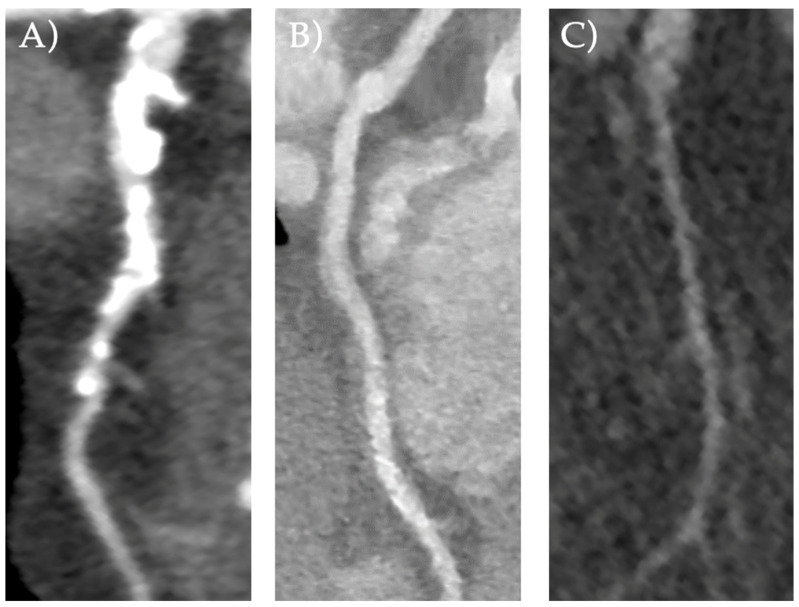
Examples of artefacts due to: (**A**) calcific bloom; (**B**) motion; (**C**) noise.

Endpoints. The primary imaging endpoint was the proportion of CTCA with suboptimal image quality and the reasons for non-evaluability. The primary clinical endpoint was the proportion of interpretable proximal segments within the suboptimal studies. The imaging outcome of CTCA image quality was evaluated based on a Likert score ranging from 1 to 4 to grade interpretability [11]: 1 = uninterpretable, impaired image quality limited by excessive noise or poor vessel wall definition; 2 = poor, greatly reduced image quality with poor vessel wall definition or excessive image noise, and limitations in low-contrast resolution remain evident; 3 = good, minimal impact of image noise, and limitations of low-contrast resolution and vessel margin definition are minimal; 4 = excellent, good attenuation of vessel lumen and delineation of vessel walls, relative image noise is negligible, and coronary wall definition and low-contrast resolution are well maintained. A score of 1 or 2 represented a suboptimal study. Results were reported at a per-patient level (i.e., if a single coronary segment was of reduced quality, the whole study was considered suboptimal). Figure 1 depicts coronary arteries from our cohort of varying evaluability as examples of diagnostic and suboptimal classifications. The reason for a suboptimal CTCA was recorded as either due to calcification obscuring lumen assessment, motion artefacts, or noise artefacts (Figure 2). 

For the primary clinical endpoint, studies that were deemed suboptimal were then individually analysed by two observers. These observers aimed to determine the evaluability of the left main coronary artery (LM) and proximal segments of the left anterior descending (LAD), left circumflex (LCx), and right coronary (RCA) arteries of CTCAs deemed suboptimal at an overall patient level. If a proximal segment was considered non-interpretable, the reason was recorded as either due to calcification obscuring lumen assessment, motion artefacts, or noise artefacts, with the single most interfering factor recorded. CAD burden assessment was carried out by quantifying plaque using the Coronary Artery Disease Reporting and Data System (CAD-RADS).

Statistical analysis. Graphpad Prism 10.0.2 (Graphpad Software, La Jolla, CA, USA), R (The Foundation for Statistical Computing; version 4.3.1) and RStudio (RStudio, Inc; Boston, MA, USA; version 2023.03.0) were used for statistical analysis. Continuous variables with a Gaussian distribution are presented as means ± standard deviation (SD), with ordinal variables presented as counts (percentages). Analysis of variance (ANOVA) with Bonferroni correction was performed across groups for continuous variables, and chi-squared tests with Fisher’s exact *t* test correction were performed across groups for ordinal variables. Predictors of a suboptimal CTCA for each patient and CTCA variable were analysed using unpaired two-tailed *t* tests. Univariable and multivariable logistic regression modelling was performed between BMI and suboptimal CTCA, with the multivariate logistic regression model incorporating the following variables: BMI ≥ 40, BMI 25–39.9, age, male sex, diabetes, heart rate, and coronary artery calcium score (CACS). A two-tailed *p*-value of < 0.05 was regarded as statistically significant.

## 3. Results

**Figure 3:**
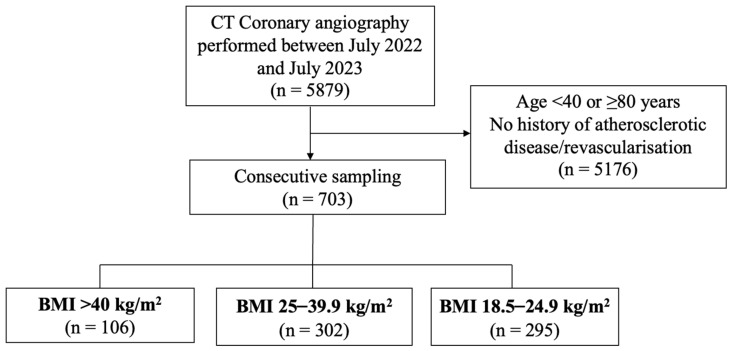
Diagrammatic representation of recruitment of participants. CT—Computed tomography, BMI—Body Mass Index.

Baseline characteristics. A total of 703 participants were included in the study. The mean age of participants was 59 ± 10 years, the mean BMI was 30.7 ± 9.6 (range: 18.6–72.4), and 47% were men. These included 295 participants with a BMI of 18.5–24.9, 302 participants with a BMI of 25–39.9, and 106 participants with a BMI ≥ 40 (Figure 3). Detailed baseline clinical characteristics and CTCA characteristics are outlined in Table 1. Statistically significant differences across BMI groups were present in the proportion of men, total diabetes mellitus (DM), type 2 DM (T2DM), hypertension, smoking, and statin use, as well as in the mean age, contrast volume used, metoprolol dose administered, and radiation dose.

**Figure 4:**
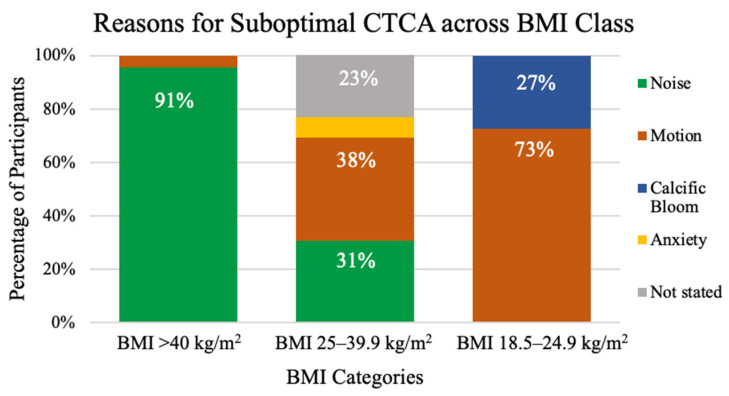
Distribution of reasons for a suboptimal CTCA within each BMI class. BMI—Body Mass Index.

Image quality. Of the 703 participants, 657 CTCAs (93.5%) were of diagnostic quality, and 46 CTCAs (6.5%) were of suboptimal quality. Participants with a CTCA of suboptimal image quality had a statistically significantly increased BMI (Supplementary Table S1). The proportion of suboptimal CTCAs was similar in participants with a BMI of 18.5–24.9 (11/295, 3.7%) and 25–39.9 (13/302, 4.3%) (*p* = 0.97). A significantly higher proportion of suboptimal CTCA rates was noted in patients with a BMI ≥ 40 (22/106, 21.5%, *p* < 0.01). The distribution of BMI across each subjective CTCA image quality grade is displayed in Supplementary Figure S1 BMI was weakly correlated with CTCA interpretability (r = −0.32 (95% CI [−0.38, −0.25], *p* < 0.01). In multivariable logistic regression analysis, independent predictors of suboptimal studies were a BMI ≥ 40 (OR 9.20, 95% CI [7.13, 11.91], *p* < 0.01), being diabetic (OR 1.90, 95% CI [1.49, 2.42], *p* < 0.01), and increased heart rate at image acquisition (OR 1.09, 95% CI [1.08, 1.10], *p* < 0.01) (Table 2). There was no evidence of interaction between diabetes and obesity class (*p* = 0.40). The primary reason for a suboptimal CTCA is illustrated in Figure 4. Noise artefacts related to body habitus were the main reason for suboptimal study classification in participants with a BMI ≥ 40 (n = 20, 91% *p* < 0.01). Motion artefacts were the main factor interfering with CTCA image quality in participants with a BMI of 18.5–24.9 and BMI of 25–39.9 (n = 3, 75% and n = 8, 72.7%, respectively, *p* < 0.01). 

Proximal segment interpretability. The suboptimal CTCAs (n = 46) were analysed to determine whether the LM, and each proximal segment of the LAD, LCx, and RCA were of adequate image quality. Of these suboptimal studies, 163/182 proximal coronary artery segments (90%) were evaluable, including 96% (42/44), 87% (40/46), 87% (40/46), and 89% (41/46) of LM, proximal LAD, proximal LCx, and proximal RCA, respectively (Supplementary Figure S2). In CTCAs deemed suboptimal at the per-patient level, the suboptimal visualisation of proximal segments was most commonly attributed to calcific blooming (12/19, 63%), followed by noise (4/19, 21%) and motion artefacts (3/19, 16%). The suboptimal proximal segments within the suboptimal CTCAs were from 7 individual participants (n = 4 participants with a BMI of 18.5–24.9, n = 2 participants with a BMI of 25–39.9, n = 1 participant with a BMI ≥ 40) (Table 3). 

Of the 46 participants with suboptimal CTCAs, 10 had subsequent cardiac investigations. This included stress echocardiography (n = 1) and ICA (n = 9). Among the nine who had ICA, four had CTCA-reported poorly visualised proximal segments. All of these patients had a BMI of 18.5–24.9 and demonstrated obstructive disease (>50% luminal stenosis) in at least one proximal coronary segment at ICA. Of the five participants with overall suboptimal CTCAs but adequate proximal segment visualisation who went on to receive ICAs, four had a BMI ≥ 40 and one had a BMI of 25–39.9 (BMI 37.5). All five of these cases demonstrated high concordance between CTCA and ICA for proximal segment evaluation. 

## 4. Discussion

In this study, we demonstrate that CTCA is a feasible investigation for the assessment of proximal coronary segments in the majority of patients with an elevated BMI ≥ 40. The majority of suboptimal studies in these patients appear to be determined by poor evaluability of the more distal segments or side branches. 

Our results reveal a low prevalence of CTCAs with suboptimal image quality, with an overall incidence of 6.5%. This is consistent with other studies [12]. Most of the literature examining the feasibility of obtaining diagnostic CTCAs from obese patients focuses on analysing the efficacy of emerging scanner technology or reducing radiation protocols in this population. Although the majority of these studies affirm that these new techniques can still successfully obtain diagnostic-grade images in people with obesity, little is discussed about the relationship between increasing bodyweight and CTCA interpretability [13]. Mangold et al. identified that a diagnostic CTCA can be obtained irrespective of BMI, but image quality was reduced in those with a BMI ≥ 40, although this BMI category had only 24 participants [14]. We used BMI as a measure of obesity, as it is the most widely used anthropometric measure, and readily calculable from clinical reports. Chest wall thickness is an alternative parameter which has been utilised elsewhere [15]. The use of other adipose metrics, such as waist circumference or body fat percentage, on image quality have not been reported. 

Obesity is typically associated with noise artefacts; as BMI (specifically, chest wall size) increases, there is an attenuation in the x-ray signal and thus a reduction in the image quality. Our study was consistent with this finding, as noise was a more prevalent reason for suboptimal CTCA acquisition in the BMI ≥ 40 group compared to lower BMI categories. The prevalence of noise artefacts as the cause of a suboptimal CTCA in those affected by obesity within the current literature is broad and has been reported to range between 30 and 78% of coronary segments [16]. Additionally, our results showed a far lower overall motion artefact rate, which may indicate better respiratory compliance, or better image reconstruction techniques with ongoing advances in CTCA image acquisition. Nonetheless, while artefacts clearly affect image quality, this may not always have clinical impact, as it is the smaller calibre (and usually more distal) vessels (which are less frequently affected by atherosclerosis and subtend a smaller area of myocardium) whose image quality is typically reduced [17].

Crucially, however, the high interpretability of the proximal coronary artery segments of CTCAs that were deemed suboptimal, demonstrated by our study, carries great clinical utility, as these segments are most often affected by coronary atherosclerosis, are culprit lesions in the majority of acute coronary syndromes (ACS), and supply a larger amount of subtended myocardium [18]. Intravascular imaging techniques to assess proximal plaque as a surrogate measure for plaque burden have found high consistency with CTCA-derived plaque burden [18]. It has also been demonstrated that proximal plaque volume assessment on CT is more reliably and rapidly identified compared with distal segments [19]. Consequently, not only should clinicians be reassured of the feasibility of CTCA in obese patients, but also recognize that even suboptimal scans carry important prognostic information and should not be discounted. We noted only one patient with a BMI ~69 as the only case where proximal segments were wholly non-evaluable due to noise artefacts. The remainder of the individuals from lower BMI groups had unpredictable reasons for non-evaluability, such as calcific blooming and motion artefacts. Nonetheless, clinicians should exercise clinical judgement to decide whether proximal segment analysis in their patients with suboptimal CTCAs is sufficient to inform risk, or if invasive measures (with their own associated risks) are required to confidently examine the entire coronary anatomy. As CTCA is generally used for individuals with suspected CAD, if there remains a suspicion of obstructive CAD causing symptoms, further invasive assessment may be needed to confirm a diagnosis. However, the use of a CTCA in an obese patient with a new diagnosis of heart failure with a severely reduced ejection fraction, for example, can provide confidence that CAD is not the aetiology in question, and can avoid the need for unnecessary invasive risk. 

Our results also demonstrated a relatively small proportion of individuals referred for an ICA following an inconclusive CTCA (9/46, 20%). This may suggest that most inconclusive CTCAs were still able to provide meaningful clinical decision-making information for the referrer. The patients with a suboptimal CTCA, including their proximal segments, that proceeded to an ICA, all had a BMI < 40. The reasons for suboptimal visualisation related to calcification/noise, and each of these patients were found to have obstructive CAD. This finding suggests that the suboptimal visualisation of proximal segments in CTCAs is often due to a high plaque burden, which is independent of body habitus. The approach of opportunistic screening as a gatekeeper for ICA has been similarly demonstrated within the pre-transcatheter aortic valve implantation population, a group in whom a high coronary calcification burden, and subsequently a high suboptimal CTCA rate, is expected. CTCA demonstrates an excellent negative predictive value with no false negatives for obstructive CAD when proximal segments are evaluable [20]. Interestingly, we noted high concordance between CTCA and ICA proximal segment stenosis severity in the patients with a BMI ≥ 40, all of whom had CTCAs that were poorly visualised due to motion/noise. While not within the scope of this study, it is interesting to note a lesser calcific burden in obese patients, and a further evaluation of plaque composition based around body fat distribution may be of interest. 

The value of CTCA as a method of CAD assessment is manifold. The combination of improving CT scanner technology and an ever-increasing obese population has resulted in an increased paradigm shift towards non-invasive methods of CAD assessment, away from traditionally invasive measures, in select patients. Latest-generation CT scanners incorporating photon counting detectors are able to produce better image quality at comparable radiations, with reduced calcific bloom artefacts from both calcified plaque and prior coronary stents [21]. Single photon emission computer tomography–myocardial perfusion imaging has shown diagnostic quality in over two-thirds of individuals with a BMI ≥ 40 and can be an alternative investigation; however, this is at the expense of significantly greater radiation exposure. Other ultrasound-based stress imaging techniques are also limited in sensitivity due to body habitus interference. In chest pain assessment and risk stratification, CTCA is the recommended first diagnostic test in several guideline recommendations [22]. The SCOT-HEART trial also demonstrated improved atherosclerotic plaque detection compared with functional assessment, which resulted in better upstream medical management and improved prognosis [23]. CTCA also provides several CAD metrics beyond stenosis to inform MACE risk that are of value in the obese population, such as epicardial adipose tissue (EAT) and coronary plaque compositional information. Obese patients have been found to have greater proximal coronary plaque volumes, including non-calcified plaque phenotypes, which are a higher risk phenotype that may be missed with lumenography [19,24]. A more nuanced understanding of the association of CAD with fat distribution and plaque phenotype through safe and widely accessible means may be vital in accurately understanding the effects of adiposity on cardiovascular disease. 

ICA in obese individuals is linked to higher complication rates including suboptimal vessel visualisation, increased rates of bleeding and prolonged hospital stays (due to vascular access difficulty), and increased radiation exposure to patients and staff to combat image noise [8,25]. With the rising prevalence of obesity, it is crucial to minimise the unnecessary exposure of these individuals to procedures where they face an increased risk of complications. Our finding of a low rate of ICA necessity highlights the strength of CTCA as a gatekeeper for CAD, ideally reserving ICA for predominantly therapeutic purposes in the stable CAD setting. 

Limitations. We acknowledge several limitations in this study. Firstly, the retrospective nature has inherent bias, and while the number of enrolled patients is small, it is one of the larger obesity-associated image quality studies. We ensured the consecutive sampling of patients with minimal exclusion criteria to try and limit selection bias. One advantage is the high proportion of those with a BMI ≥ 40 that we have been able to capture, which other studies have lacked significant representation for. Furthermore, our study may not capture the full spectrum of patients, as those deemed at higher risk may have been referred directly for invasive testing rather than undergoing CTCA. Since the role of CTCA is firmly established in low- to intermediate-risk patients, extrapolating the study’s conclusions to higher-risk patients may not be as applicable or representative. The cases were also drawn from a single clinical practice rather than registry data, potentially introducing location bias. We specifically employed a subjective 4-point Likert score to emulate the subjective nature of CTCA assessment in real-world scenarios, and these subjective CT image quality assessments correlate well with objective measures [26]. Further evaluation with metrics such as signal-to-noise ratio are of value, but not necessarily pragmatic in a routine clinical context, and the aim of this study was to understand current reporting practices rather than to focus on the optimisation of image acquisition. 

## 5. Conclusions

Obesity is a recognised and significant contributor to CAD, but the optimal mode of assessment remains uncertain due to concerns of poorer image quality. Despite a modestly higher rate of suboptimal studies when patient BMI exceeds 40, our study demonstrates that important prognostic information, such as proximal epicardial coronary stenosis, is still largely obtainable in the vast majority of patients, regardless of their BMI. This has potential clinical impact for the widespread employment of CTCA as a non-invasive anatomical test for the diagnosis of CAD.

## Figures and Tables

**Table 1:** Baseline clinical demographics and CTCA parameters across BMI categories.

BMI Category	BMI > 40 (Group 1) (n = 106)	BMI 25–39.9 (Group 2) (n = 302)	BMI 18.5–24.9 (Group 3) (n = 295)	*p*-Value
Male	32 (30.2)	163 (54.0) *	138 (46.8) ᶲ	<0.01
Age, years	54.2 ± 8.1	58.4 ± 9.7 *^#^	60.6 ± 11.0 ᶲ	<0.01
BMI, kg/m^2^	48.6 ± 6.8	32.2 ± 4.1 *^#^	22.7 ± 1.7 ᶲ	<0.01
Diabetes	13 (12.3)	44 (14.6) ^#^	23 (7.8)	0.03
Dyslipidaemia	54 (50.9)	154 (51.0) ^#^	152 (51.5)	0.91
Hypertension	41 (38.7)	158 (52.3) *	115 (39.0)	<0.01
Smoking	35 (33.0)	118 (39.1) ^#^	87 (29.5)	0.05
Family history of premature CAD	37 (34.9)	88 (29.1)	94 (31.9)	0.51
AF	4 (3.8)	9 (3.0)	10 (3.4)	0.91
Statin	29 (27.4)	94 (31.1) ^#^	63 (21.4)	0.03
Heart rate, bpm	62.0 ± 12.3	60.9 ± 11.36	60.3 ± 13.8	0.2
Contrast Dose, mL	66.2 ± 14.0	60.6 ± 8.5 *^#^	49.8 ± 5.6 ᶲ	<0.01
Metoprolol IV, mg	10.5 ± 13.3	7.7 ± 10.0 *^#^	4.5 ± 7.0 ᶲ	<0.01
Radiation dose, mSv	4.0 ± 1.8	4.0 ± 2.2 ^#^	1.9 ± 1.2 ᶲ	<0.01
CACS	59.5 ± 218.7	106.3 ± 315.0	92.1 ± 286.7	0.48

Continuous variables presented as means ± standard deviation (SD). Ordinal variables presented as count (percentage). Two- and three-way group comparison by *t*-test, ANOVA, or chi-squared test, as appropriate. *p*-values column from table represents Group 1 vs. 2 vs. 3. * indicates Group 1 vs. 2 *p* < 0.05; ^#^ indicates Group 2 vs. 3 *p* < 0.05; ᶲ indicates Group 1 vs. 3 *p* < 0.05. BMI—Body Mass Index, CAD—Coronary Artery Disease, AF—Atrial Fibrillation, bpm—beats per minute, mL—millilitres, IV—Intravenous, mg—milligrams, mSv—millisievert, CACS—Coronary Artery Calcium Score.

**Table 2:** Odds ratios for multivariable logistic regression model for BMI association with suboptimal CTCA image quality.

Predictor	Odds Ratio [95% CI]	*p*-Value
BMI 18.5–24.9 kg/m^2^	Reference	Reference
BMI > 40 kg/m^2^	9.20 [7.13, 11.91]	<0.01
BMI 25–39.9 kg/m^2^	1.26 [0.99, 1.60]	0.06
Age, years	0.99 [0.99, 1.01]	0.84
Male	0.81 [0.66, 0.99]	0.04
Diabetes	1.90 [1.49, 2.42]	<0.01
Heart Rate, bpm	1.09 [1.08, 1.10]	<0.01
CACS	1.00 [1.00, 1.00]	<0.01

CI—Confidence Interval, BMI—Body Mass Index, bpm—beats per minute, CACS—Coronary Artery Calcium Score.

**Table 3:** Individual clinical and CTCA data for the 7 participants with suboptimal CTCAs who also had 1 or more suboptimally visualised proximal coronary segment.

	Age	Sex	BMI	HR	Evaluable LM	Evaluable LAD	Evaluable LCx	Evaluable RCA	Reason for Non-Evaluability
1	40	M	68.6	55	No	No	No	No	Noise
2	74	M	32.7	61	No	No	No	No	Calcific bloom
3	66	F	26.4	65	Yes	Yes	No	Yes	Calcific bloom
4	74	M	23.6	63	Yes	No	No	No	Calcific bloom
5	76	F	23.5	85	N/A	No	No	No	Motion
6	76	F	23.3	72	Yes	No	Yes	Yes	Calcific bloom
7	72	M	21.9	79	Yes	No	No	No	Calcific bloom

BMI—Body Mass Index, HR—heart rate, LM—left main coronary artery, LAD—left anterior descending artery, LCx—left circumflex artery, RCA—right coronary artery, M—Male, F—Female, N/A—not applicable. Patient BMI categories are marked as follows: brown (>40, i.e., patient 1), yellow (25–39.9, i.e., patient 2 and 3) and green (18.5–24.9, i.e., patients 4–7).

## Data Availability

De-identified data are available.

## Supplementary Materials

The following supporting information can be downloaded at https://www.mdpi.com/article/10.3390/jcdd11120400/s1, Table S1: Baseline clinical characteristics of suboptimal and diagnostic participants; Figure S1: Box and whisker plot conveying the distribution of BMI within each CTCA image quality score; Figure S2: Evaluability of LM and proximal segments of LAD, LCx, and RCA.
