# Supplementary material for: Computed Tomography Coronary Angiography Is Feasible and Reliable for Proximal Coronary Segment Interpretation in Patients with Elevated Body Mass Index
Source: J Cardiovasc Dev Dis. 2024 Dec 11;11(12):400. doi: 10.3390/jcdd11120400 (PMC11678149; doi:10.3390/jcdd11120400)
Supplement: Supplementary file 1 [file jcdd-11-00400-s001.zip › jcdd-3332203-supplementary.pdf]

Table S1: Baseline clinical characteristics of suboptimal and diagnostic participants

CTCA Interpretability	Suboptimal (n=46)	Diagnostic (n=657)	P-value
Male, n (%)	18 (39.1)	314 (47.7)	0.29
Age, y, mean ± SD	56.6 ± 0.4	58.8 ± 10.1	0.15
BMI, kg/m², mean ± SD	40.3 ± 0.3	30.0 ± 8.6	**<0.01**
Diabetes, n (%)	8 (17.4)	72 (11.0)	0.22
Dyslipidaemia, n (%)	28 (60.9)	332 (50.5)	0.22
Hypertension, n (%)	25 (54.4)	289 (44.0)	0.22
Smoking, n (%)	15 (32.6)	225 (34.3)	0.87
Family history of premature CAD, n (%)	13 (28.3)	206 (31.4)	0.74
AF, n (%)	6 (13.0)	17 (2.6)	**<0.01**
Statin, n (%)	18 (39.1)	168 (25.6)	0.056
Aspirin, n (%)	9 (19.6)	65 (9.9)	**0.04**

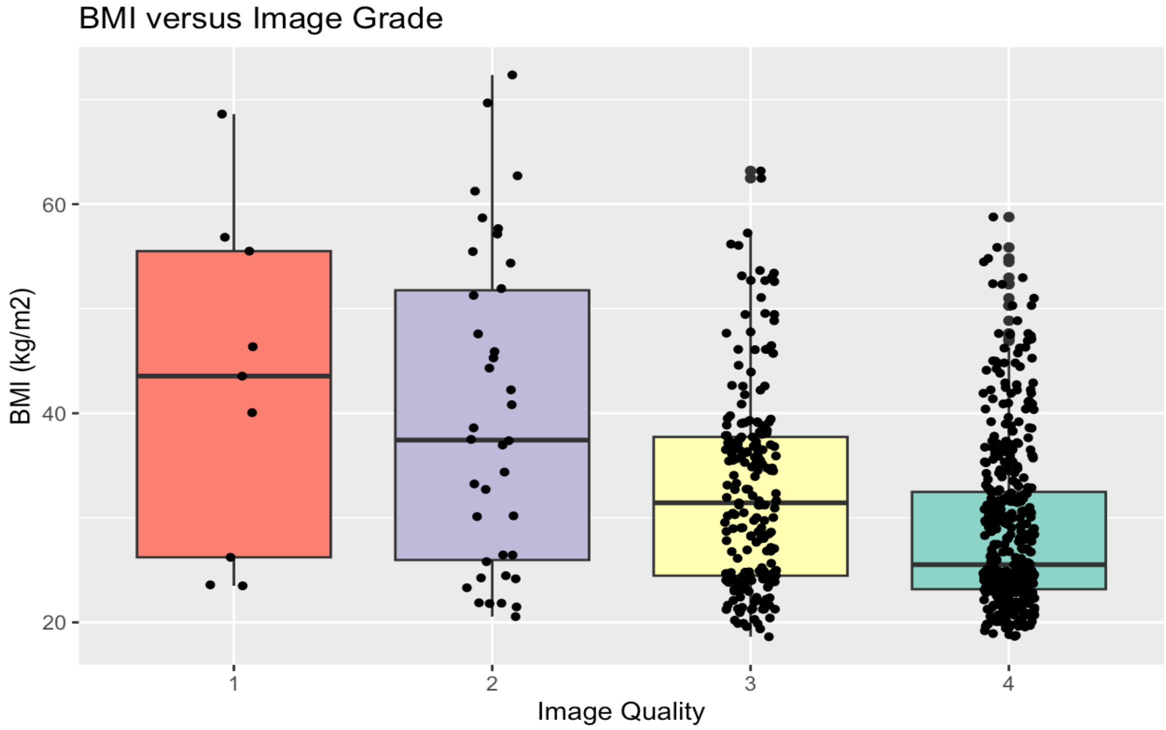

Figure S1: Box and whisker plot conveying the distribution of BMI within each CTCA image quality score. Each dot represents a participant's BMI (y-axis) within their respective CTCA image quality grade:
1=red: uninterpretable, impaired image quality limited by excessive noise or poor vessel wall definition.
2=purple: poor, greatly reduced image quality with poor vessel wall definition or excessive image noise, limitations in low contrast resolution remain evident
3=yellow: good, minimal impact of image noise, limitations of low contrast resolution and vessel margin definition are minimal
4=green: excellent, good attenuation of vessel lumen and delineation of vessel walls, relative image noise is negligible, coronary wall definition and low contrast resolution well maintained.
A score of 1 or 2 represented a suboptimal study. Results were reported at a per-patient level (i.e., if a single coronary segment was of reduced quality, the whole study was considered suboptimal).

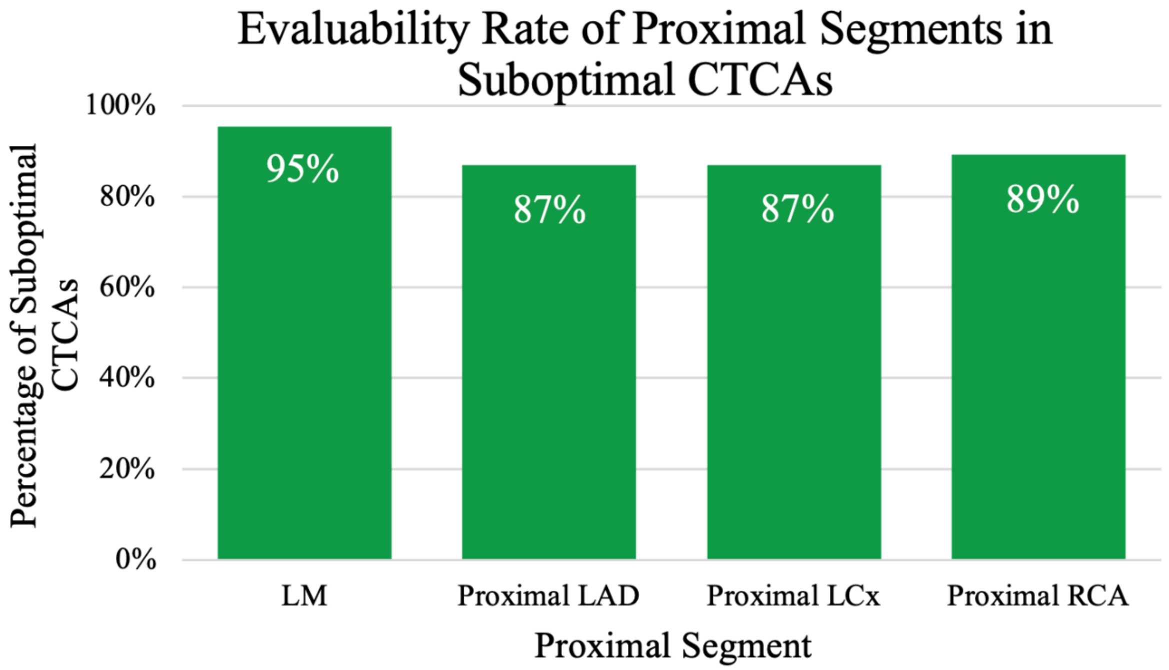

Figure S2: Evaluability of LM and proximal segments of LAD, LCx, and RCA.
